# Remarks on Strangulated Umbilical Hernia, with a Case

**Published:** 1847-02

**Authors:** A. J. Wedderburn

**Affiliations:** Professor of Anatomy in the Medical College of Louisiana


*Remarks on Strangulated Umbilical Hernia, with a Case*, by A. J. Wedderburn, M. D., Professor of Anatomy in the Medical College of Louisiana.—Strangulated umbilical hernia, being an affection of very rare occurrence in the adult male, it is deemed proper to report the following case for which a successful operation has been performed.

A negro man of 300 pounds weight, aged 31 years, the property of Dr. Slone, residing about two miles below New Orleans, states that he has always had an umbilical hernia, easily reducible, and about the size of a hen's egg—was attacked with a pain in the abdomen—found the tumour enlarged—attempted its reduction, but failed. I saw this case about 12 hours after the strangulation occurred, and found the tumour about the size of a child's head, very tense and elastic. About three hours before I saw the case Dr. Slone had used the various means recommended for reduction in such cases, and on my visiting the case with him, we further endeavoured for the space of an hour, to effect it by taxis, and to aid in the attempt we administered tartar emetic, tobacco injections, large injections of cold water with a hydrostatic injecting tube, and applied ice and sulphuric ether to the tumour. These efforts having failed, we determined upon an operation as the only means calculated to afford the patient a chance for his life. A short time previous to the operation, whilst making forcible taxis, a considerable portion of gas contained in the incarcerated bowl escaped with a gurgling sound, and the tumour seemed to subside so rapidly under my hand, that I was induced to believe that I had succeeded in the reduction, but in a few minutes was convinced that the intestine could not be returned, or even the escape of more of the gas effected. By this effort the tumour was reduced so much by the escape of air from the intestine, and the tension consequently so much relieved, as to lessen the danger of wounding the intestine in dividing the integuments. We informed the patient of the necessity of an immediate operation, to which he somewhat objected, and only consented after we had allowed him to make an effort at reduction himself, which he continued for something like ten minutes, using all the while considerable and well directed force, for he had returned the intestine so often himself when the hernia was smaller and reducible, that he had acquired quite a degree of dexterity in the matter.

*Operation.*—An incision was made upon the top of the tumour three or four inches in length through the skin in a vertical direction, so as to expose the superficial fascia, which was considerably condensed. A small opening was made through the fascia at the lower part of the wound, by light and careful touches with a scalpel, into which a director was introduced, and carried to the upper part of the first incision, and the fascia divided to correspond with the same, with a probe pointed bistoury. I then introduced my finger between the peritoneal sac and the superficial fascia, and carrying it in every direction, easily effected the separation of the two membranes. Finding there was not room to work in, the wound was enlarged by extending it about an inch above, and more than an inch below,—this enabled me to turn aside the integuments, and obtain a good view of the sac, which contained, floating in a nearly transparent fluid, a large portion of the omentum, and as well as I could determine, about fifteen inches of the small intestinal tube, which appeared to be perfectly black. A small opening was next made in the lower part of the sac, by seizing up a small portion of it with a pair of artery forceps, and carefully cutting in a horizontal direction. After the escape of about four or five ounces of fluid, a director was introduced, and the sac divided upon it about four inches. In a few moments the intestine, which presented the dark colour before mentioned, began to assume a red appearance, from the action of the air acting upon the blood contained in its vessels, through the coats of the intestine; from which circumstance, and also from a close examination, I was convinced that the gut retained its integrity, and at once endeavoured to return it by an attempt to kneed in a small portion at a time;—but in my efforts I not only failed, but additional portions of the omentum and intestine were forcing themselves from the abdomen. In order then, that the stricture could be arrived at without endangering the gut, it became necessary to extend the incisions, not only in the integuments nearly to the base of the tumour, but also in the peritoneal sac. The finger was then forced into the umbilical ring, and a probe pointed bistoury passed by its side, with which the structure was divided at the lower part about the fourth of an inch. An attempt was again made to return the bowel, but without success, when a division of the ring was made at the upper part, less than a fourth of an inch in extent, by which means I was enabled to return the bowel by pushing in a small portion at a time. After returning the intestine, the omentum was returned, and spread out in front of the small intestines, as well as it could be done by the introduction of my finger into the cavity of the abdomen for this purpose.

After returning the contents of the sac, the blood was sponged from the wound, then leaving it open for the space of about 15 minutes, in order that the oozing from the divided vessels might be entirely arrested, the edges of the wound were brought in contact, and three interrupted sutures applied. The skin, which was very loose, was gathered up like a bag, with the sack contained within, but not included in the ligatures, and a graduated compress applied, with the hope that the mass would, if healed, be a barrier to a further protrusion, and effect a radical cure.

The operation was performed on the 23d of September last, and the patient recovered without a bad symptom. Small doses of calomel and opium were administered during four or five days. In ten days after the operation the patient walked across the room and is now entirely well, a radical cure having been effected.

As the operation just described differs somewhat from those recommended by different surgeons, I have thought proper to give below a few extracts, with some general remarks on the subject of exomphalos.

In Cooper's Surgical Dictionary we find the following: "In consequence of the great fatality of the usual operation for the exomphalos, I think the plan suggested and successfully practiced by Sir A. Cooper in two instances, should always be adopted when the tumour is large and free from gangrene; a plan that has also received the high sanction of that distinguished anatomist and surgeon, Professor Scarpa. (*Traité des Hernies, p.* 362.) Perhaps I might safely add, that when the parts admit of being reduced, without laying open the sack, this method should always be preferred. It consists in making an incision just sufficient to divide the stricture, without opening the sack at all, or, at all events, no more of it than is inevitable."

In umbilical hernia, of not a large size, Sir A. Cooper recommends the following plan of operating: "As the opening into the abdomen is placed towards the upper part of the tumour, I began the incision a little below it, that is, at the middle of the swelling and extended it to its lowest part. I then made a second incision at the upper part of the first, and at right angles with it, so that the double incision was in the form of the letter T, the top of which crossed the middle of the tumour. The integuments being thus divided, the angles of the incision were turned down, which exposed a considerable portion of the hernial sack. This being then carefully opened, the finger was passed below the intestines to the orifice of the sack at the umbilicus, and the probe-pointed bistoury being introduced upon it, I directed it into the opening at the navel, and divided the linea alba downwards to the requisite degree, instead of upwards as in the former operation. When the omentum and intestine are returned, the portion of integument and sack which is left, falls over the opening at the umbilicus, covers it, and unites to its edge, and thus lessens the risk of peritoneal inflammation, by more readily closing the wound.

In Gibson, page 128, we find the following: "Strangulated umbilical hernia very frequently proves fatal, as much from disorder of the intestinal function as from the strangulation. When the usual remedies fail, an operation should be resorted to. This may be done in the following way. An incision, several inches long, is made very cautiously through the integuments and superficial fascia, when the sack, if not absorbed, as it often is, will appear. Into this a small opening should be made, from which fluid in considerable quantity generally issues. The opening may then be enlarged, and a finger carried upwards between the omentum and intestine as high as the umbilical ring. Upon the finger a bistoury is next to be carried through the linea alba, to the extent of an inch, which, in most cases will relieve the stricture sufficiently to enable the operator without much difficulty to restore the parts to their former situation.

"Dr. Physick has proposed, in strangulated umbilical hernia, to make a crucial incision through the integuments, as far as the neck of the sack, then open the sack at its upper part to an extent sufficient to enable the operator to examine its contents, and reduce them, if possible, without dilating the umbilical ring. Should the latter expedient, however, become necessary, the stricture must be divided on the outside of the sack. After the omentum and the intestine are restored to the abdomen, a ligature should be drawn around the neck of the sack, with a view of closing the cavity and obviating peritoneal inflammation. The late Dr. Wistar once performed the operation with success. In the case of a Mrs. N., a very respectable Jewish lady, I performed a similar operation about fifteen years ago. The tumour, however, was as large as a child's head, and had been strangulated several days before I saw the patient, and, on this account, the operation did not succeed. The patient, too, was advanced in years, extremely corpulent, and had long suffered from derangement of the functions of the stomach and intestines. Under these circumstances, no operation, probably, would have answered the purpose, even if performed in the very commencement of strangulation."

In Lawrence, on ruptures, we find the following:

"The greatest practical writers have strongly represented the frequent fatality of the operation for strangulated exomphalos; and the results of my own experience coincide entirely with their statements. I have, indeed, operated successfully on a large intestinal exomphalos, containing several convolutions of small intestine, of a bright red colour, without any omentum. in a fat woman advanced in years; but the majority of cases, in which I have either operated myself, or seen the operation done by others, have ended fatally.

"Perhaps this fatality may be in some degree explained by considering that the exomphalos is most frequently in fat gross subjects, unfavourable for operations; that general intestinal disorder either exists with rupture, or is speedily produced by it; and that irritation and inflammation are readily propagated to the stomach, which is close to the umbilicus."

In the same work we find the following: "The great fatality of the ordinary operation for exomphalos makes it advisable that we should employ every precaution calculated to diminish subsequent irritation and inflammation. Hence it would be proper to adopt, especially if the tumour exceed a moderate size, the mode of operating which is applicable to large inguinal herniæ; in which the tendon is divided without opening the sack; or the latter part is only cut sufficiently to allow the division of the structure. This will permit the return of the parts if they are not adherent; and if adhesion should have formed, the immediate cause of danger, the strangulation is removed. The approximation of the sides of the wound by sutures, or adhesive plaster, will prevent the occurrence of inflammation in the tumour. The practicability of this mode of operating in umbilical ruptures is fully proved by two cases recorded in the work of Sir A. Cooper, and the successful termination of both instances proved the judgment and sagacity which had suggested that peculiar treatment."

From the concluding remark of the foregoing paragraph it will be perceived, that great credit is awarded Sir A. Cooper for his success in operating without dividing the peritoneal sack.

Too much importance is no doubt attributed to wounding the peritoneum, and this opinion is well established by the recorded cases in Churchill, of Cæsarean operations, in which mothers were saved. We must look to the anatomy of the umbilical ring for the true explanation of the fatality attending operations for exomphalos: and I think we will learn, that to be more successful, we should apply our different means for reduction in quick succession, so that failing in all but the knife, we may resort to it the sooner. If we introduce a finger into the umbilical ring, through which a protrusion which is reducible has taken place, we will at once be convinced from the firmness and unyielding nature of its walls, that a strangulation occurring at this point, would necessarily result in a disorganization of the tissues much more rapidly, than when it takes place at the internal inguinal ring, which is the mouth of a sac (the tunica vaginalis communis) continuous with the fascia transversalis, a thin elastic membrane, capable of considerable distension under any circumstances. Lawrence relates a case of a lady forty-eight years of age, terminating fatally in seventeen hours from the commencement of the strangulation.

Different methods have been given for the division of the stricture—such as dividing the linea alba downwards, to the extent of an inch; its division upwards has also been recommended, and Velpeau recommends, that several slight nicks be made at different points of the ring. Dividing the ring downwards to the extent of an inch, would make so large an opening as to present an obstacle in the way of a radical cure. In the case related in the first part of this paper, the ring was divided about a quarter of an inch at the lower part, and this division not being sufficient, a mere nick at the upper part relieved the stricture. In cases where much cutting would be necessary to relieve the stricture, the plan proposed by Velpeau, enables us to relieve the stricture, without augmenting much the size of the ring, as effectually, as by a single long incision in any direction.

The only cases that I have been enabled to find recorded, in which this operation has been successfully performed, are two cases by Sir A. Cooper, one by Mr. Lawrence, one by Mr. Walker of Hurtsperpont, one by Velpeau. All the operations mentioned, were upon females. I have not been able to learn, whether the successful operation mentioned before, by Dr. Wistar, was upon a male or female.—*New Orleans Med. and Surg. Journ.*

